# *De novo* Sequencing, Characterization, and Comparison of Inflorescence Transcriptomes of *Cornus canadensis* and *C. florida* (Cornaceae)

**DOI:** 10.1371/journal.pone.0082674

**Published:** 2013-12-27

**Authors:** Jian Zhang, Robert G. Franks, Xiang Liu, Ming Kang, Jonathan E. M. Keebler, Jennifer E. Schaff, Hong-Wen Huang, Qiu-Yun (Jenny) Xiang

**Affiliations:** 1 State Key Laboratory of Systematic and Evolutionary Botany, Institute of Botany, Chinese Academy of Sciences, Beijing, P.R. China; 2 Department of Plant and Microbial Biology, North Carolina State University, Raleigh, North Carolina, United States of America; 3 CAS Key Laboratory of Plant Resource Conservation and Sustainable Utilization, South China Botanical Garden, Chinese Academy of Sciences, Guangzhou, P.R. China; 4 Bioinformatics Analyst and Consultant Genomic Sciences Laboratory, North Carolina State University, Raleigh, North Carolina, United States of America; Wuhan Botanical Garden, Chinese Academy of Sciences, Wuhan, China

## Abstract

**Background:**

Transcriptome sequencing analysis is a powerful tool in molecular genetics and evolutionary biology. Here we report the results of *de novo* 454 sequencing, characterization, and comparison of inflorescence transcriptomes of two closely related dogwood species, *Cornus canadensis* and *C. florida* (Cornaceae). Our goals were to build a preliminary source of genome sequence data, and to identify genes potentially expressed differentially between the inflorescence transcriptomes for these important horticultural species.

**Results:**

The sequencing of cDNAs from inflorescence buds of *C. canadensis* (cc) and *C. florida* (cf), and normalized cDNAs from leaves of *C. canadensis* resulted in 251799 (ccBud), 96245 (ccLeaf) and 114648 (cfBud) raw reads, respectively. The *de novo* assembly of the high quality (HQ) reads resulted in 36088, 17802 and 21210 unigenes for ccBud, ccLeaf and cfBud. A reference transcriptome for *C. canadensis* was built by assembling HQ reads of ccBud and ccLeaf, containing 40884 unigenes. Reference mapping and comparative analyses found 10926 sequences were putatively specific to ccBud, and 6979 putatively specific to cfBud. Putative differentially expressed genes between ccBud and cfBud that are related to flower development and/or stress response were identified among 7718 shared sequences by ccBud and cfBud. Bi-directional BLAST found 87 (41.83% of 208) of *Arabidopsis* genes related to inflorescence development had putative orthologs in the dogwood transcriptomes. Comparisons of the shared sequences by ccBud and cfBud yielded 65931 high quality SNPs between two species. The twenty unigenes with the most SNPs are listed as potential genetic markers for evolutionary studies.

**Conclusions:**

The data provide an important, although preliminary, information platform for functional genomics and evolutionary developmental biology in *Cornus*. The study identified putative candidates potentially involved in the genetic regulation of inflorescence evolution and/or disease resistance in dogwoods for future analyses. Results of the study also provide markers useful for dogwood phylogenomic studies.

## Introduction

The dogwood genus (*Cornus* L.) belongs to the eudicot family Cornaceae in the order Cornales of the Asterids clade. It consists of approximately 55 species that are mostly evergreen or deciduous trees or shrubs, and rarely rhizomatous herbs [1]–[4]. Many dogwood species are valued in horticulture because of their spectacular blooms in the spring and brightly colored fruits, leaves, or stems in the fall or winter. A short list of the well-known examples includes the flowering dogwood *C. florida* L., the kousa dogwood *C. kousa* Hance, the cornelian cherry *C. mas* L., the bloodtwig dogwood *C.* s*anguinea* L., the red-osier dogwood *C. sericea* L. (syn. *C. stolonifera* Michx.), and the bunchberry *C. canadensis* L. f. One of the cornelian species *C*. *officinalis* Siebold & Zucc. is also highly valued in Chinese medicine and cultivated as a crop in China. Dogwoods are widely distributed in the north temperate regions extending to the tropical and subtropical areas of Asia, America, and Africa, with several species isolated in small areas of these continents [3], [5]. One striking feature of the dogwood genus is the considerable variation in morphology of inflorescences, bracts, and fruits among species [1], [5]–[7]. A number of studies on the taxonomy, phylogeny, biogeography, and morphological evolution of this genus have been recently conducted to better understand the biodiversity and evolutionary history of this economically important group [1], [3]–[15], [39]. For example, phylogenetic studies using molecular data have shown that the modern dogwood species represent descendants of four closely related evolutionary lineages that have diverged in inflorescence morphology: (1) the blue- or white-fruited dogwoods bearing large, elongated inflorescences with rudimentary bracts on the inflorescence branches, e.g., *C. sericea*, *C. sanguinea*, *C. macrophylla* Wall.; (2) the cornelian cherries with umbel-like inflorescences that are subtended by four non-petaloid involucral bracts (bracts at the base of inflorescences), e.g., *C. mas*, *C. officinalis*; (3) the big-bracted dogwoods with head-like inflorescences subtended by four or six large, petaloid, involucral bracts, e.g., *C. florida*, *C. kousa, C. nuttallii* Audubon; and (4) the dwarf dogwoods, rhizomatous herbs with small, condensed, dichasia subtended by four large, petaloid, involucral bracts, e.g., *C. canadensis*, *C. suecica* L. [1], [10]–[12]. This variation among the four closely related lineages of dogwoods provides a very useful system to study inflorescence evolution and the underlying molecular mechanisms. In particular, the genus is an excellent system for studying the origins of umbel-like and head-like inflorescences and petaloid bracts that have evolved many times during angiosperm diversification, but are poorly understood. Comparative analyses of inflorescence developmental morphology based on the dogwood phylogeny suggested that umbel-like and head-like inflorescences in *Cornus* evolved independently from elongated forms through an umbellate dichasium ancestor, and the petaloid bracts in *C. canadensis* and *C. florida* also evolved independently via different developmental mechanisms [14]–[15]. These previous phylogenetic and developmental studies provide the necessary framework for investigating the genetic basis of inflorescence evolution in the genus. However, the genus lacks sequence data at genomic scale to facilitate such investigation.

In addition to the striking evolutionary divergence in inflorescence morphology, the four major dogwood lineages also display variation in fruit type (simple vs. compound), fruit color (white, blue, black, red, purple red), growth habit (trees, shrubs, herbs), chromosome number (x = 11, 10, 9), pollination mechanism, freezing tolerance, wood anatomy, phytochemistry, as well as disease resistance [4], [16]–[17]. Of particular interest is the susceptibility of this genus to the fungal disease - dogwood anthracnose that affects some North American species and has caused serious damage and decrease of natural populations of *C. florida* throughout its range [17]–[19]. *Cornus florida* is an important ecological element of the temperate forests in eastern North America and the state flower of North Carolina and the state tree of Virginia. This species is the most vulnerable victim of this disease, while other species have shown to be resistant to the dogwood anthracnose to various degrees [17]–[19]. At present, the genetic basis and molecular mechanism for the resistance and susceptibility of dogwoods to the fungal pathogen are still unknown. It is well recognized that genome and transcriptome sequences are fundamental to genetic research of morphology. To our knowledge, there are few genomic or transcriptomic resources of any dogwood species available to the public.

**Figure 1:**
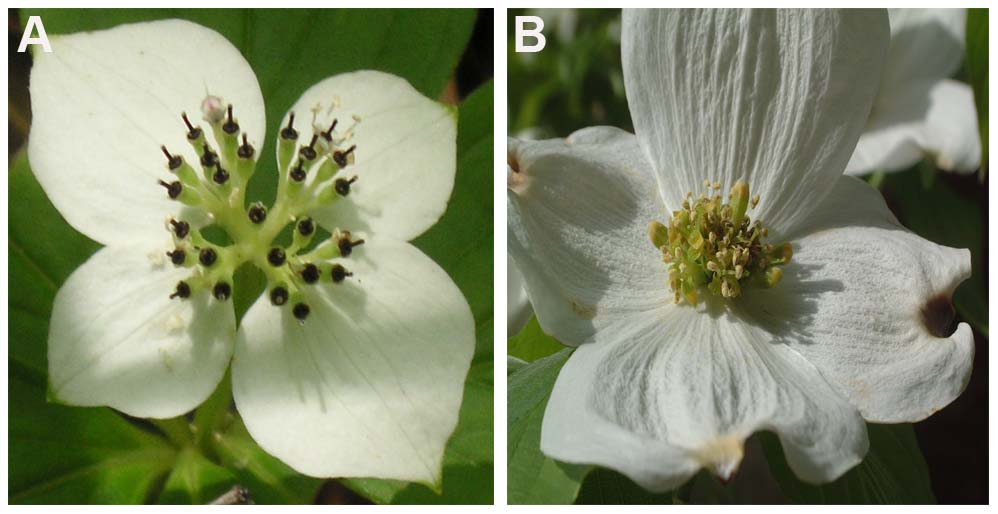
Inflorescence morphology of *Cornus canadensis* (A) and *C. florida* (B).

Transcriptome analysis provides valuable insights into genes and gene activities responsible for differences of organ morphology during the developmental processes. As demonstrated in recent studies, transcriptome analyses using next generation sequencing tools have become increasingly common to unravel genetic network regulating the development and morphology [20]–[22]. Here we applied this approach to characterize two inflorescence transcriptomes and one leaf transcriptome of *Cornus* L. to provide the first source of transcriptomic data for the genus. Comparisons between the two inflorescence transcriptomes permitted identification of a pool of potentially differential expressed genes (DEGs) between the two inflorescence types, in addition to a number of genes containing SNP markers useful for evolutionary studies. Specifically, we employed Roche 454 GS-FLX, next generation sequencing (NGS) method, for *de novo* sequencing of inflorescence transcriptomes of *C. canadensis* (referred to as ccBud hereafter) and *C. florida* (referred to as cfBud hereafter) that are from two sister lineages, the dwarf dogwoods and the big-bracted dogwoods, respectively [14] (Figure 1). The leaf transcriptome of *C. canadensis* (referred to as ccLeaf hereafter) was also sequenced using this method to maximize the coverage for transcripts of the species and to provide a background reference in identifying only the genes involved in inflorescence development. The transcriptomes were assembled *de novo* using the pool of sequencing data, and the corresponding contigs and singletons were annotated. According to gene ontology (GO) terms, homologs of genes potentially involved in flower development (including those related to inflorescence development) and stress response (including those related to disease response) were identified for each species transcriptome. Putative DEGs from each of these categories between *C. canadensis* and *C. florida* inflorescence transcriptomes were also identified based on Reads per Kilobase per Million mapped Reads (RPKM) [23]. Furthermore, we used bi-directional (or reciprocal) BLAST to determine if orthologs of *Arabidopsis* genes known to regulate inflorescence architecture and/or express in inflorescence meristem were present in the ccBud and cfBud transcriptomes. The putative DEGs are to serve as the candidates of future quantitative Real-Time PCR (qRT-PCR) and *in situ* hybridization analyses in order to identify genes contributing to the changes of inflorescence architectures in *Cornus*. Finally, genes containing high quality (HQ) single nucleotide polymorphisms (SNPs) between these two species were also identified to provide genetic markers for future phylogenomic and evolutionary ecological genomic studies that are fundamental to the conservation of dogwood biodiversity.

## Results

### Sequencing and assembly

**Figure 2:**
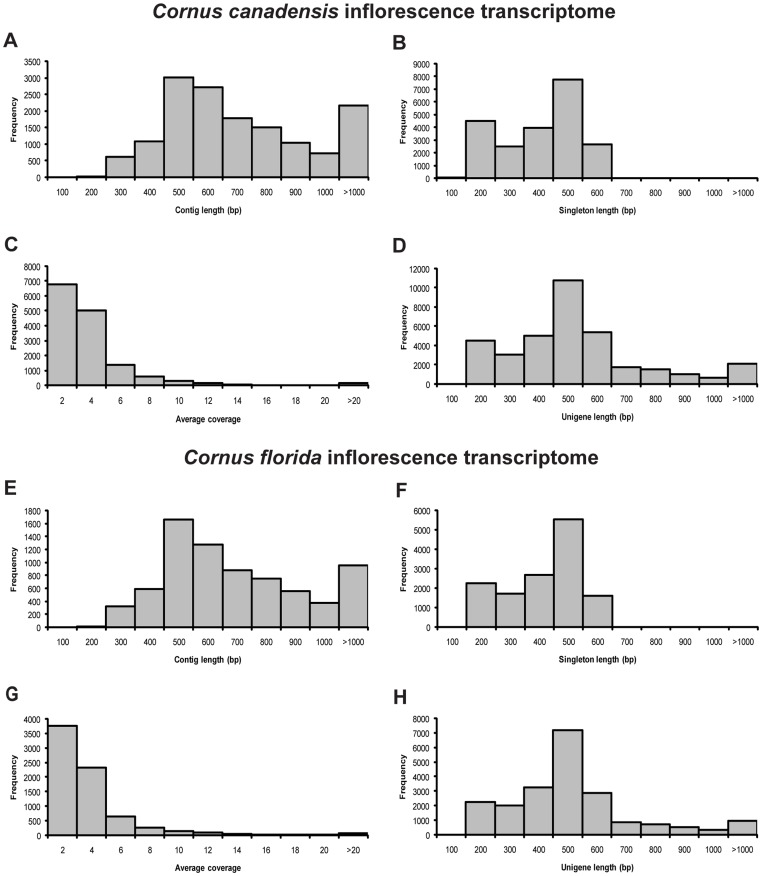
Assembly characteristics of *Cornus canadensis* inflorescence transcriptome (ccBud) and *C. florida* inflorescence transcriptome (cfBud). (A–D) ccBud and (E–H) cfBud. (A,E) Length frequency distribution of assembled contigs. (B,F) Length frequency distribution of singletons. (C,G) Average coverage frequency distribution of assembled contigs. (D,H) Length frequency distribution of unigenes.

The 454 sequencing of cDNA libraries of ccBud, ccLeaf and cfBud yielded 251799, 96245, and 114648 raw reads with average lengths of 395 bp, 317 bp and 392 bp, respectively and the most frequent length of 450–550 bp (Table 1; Fig. S1). After the sequence trimming, 251679, 85703 and 114605 HQ reads of ccBud, ccLeaf and cfBud were obtained, corresponding to 99.95%, 89.05% and 99.96% of the original raw reads (Table 1). The average length of HQ reads was 391 bp for ccBud, 322 bp for ccLeaf and 388 bp for cfBud. The most frequent length of HQ reads in all samples was also in the 450–550 bp range (Table 1; Figure S1). Through *de novo* assembly, 226675, 73775 and 98096 HQ reads of ccBud, ccLeaf and cfBud were assembled into 14656, 7574 and 7366 contigs, respectively (Table 1). The mean of average coverage of assembled contigs was assessed as five for ccBud, five for ccLeaf and four for cfBud (Table 1). And 21432, 10228 and 13844 HQ reads of ccBud, ccLeaf and cfBud were retained as singletons (Table 1). The average length of contigs and singletons was 699 bp and 358 bp for ccBud, 607 bp and 345 bp for ccLeaf, 680 bp and 369 bp for cfBud, respectively (Table 1). In total, the contigs and singletons resulted in 36088, 17802 and 21210 unigenes for ccBud, ccLeaf and cfBud, with the average length of 496 bp, 456 bp and 477 bp, respectively (Table 1). The distributions of contig, singleton, and unigene lengths and average coverage of assembled contigs for ccBud and cfBud are shown in Figure 2 and those for ccLeaf are shown in Figure S2.

**Table 1:** Summary of assembly, BLAST and annotation of sequences resulting from 454 sequencing.

	*Cornus canadensis*		*Cornus florida*
	ccBud	ccLeaf	cfBud
Raw reads	251799	96245	114648
Average length of raw reads (bp)	395	317	392
HQ reads (% of total raw reads)	251679 (99.95%)	85703 (89.05%)	114605 (99.96%)
Average length of HQ reads (bp)	391	322	388
Assembled contigs	14656	7574	7366
Mean of average coverage	5	5	4
Average length of contigs (bp)	699	607	680
Singletons	21432	10228	13844
Average length of singletons (bp)	358	345	369
Unigenes	36088	17802	21210
Average length of unigenes (bp)	496	456	477
Number of unigenes with BLAST matches (% of total unigenes)	22231 (61.60%)	12434 (69.85%)	13723 (64.70%)
Number of unigenes with GO annotation (% of total unigenes)	17229 (47.74%)	9728 (54.65%)	10694 (50.42%)

**Figure 3:**
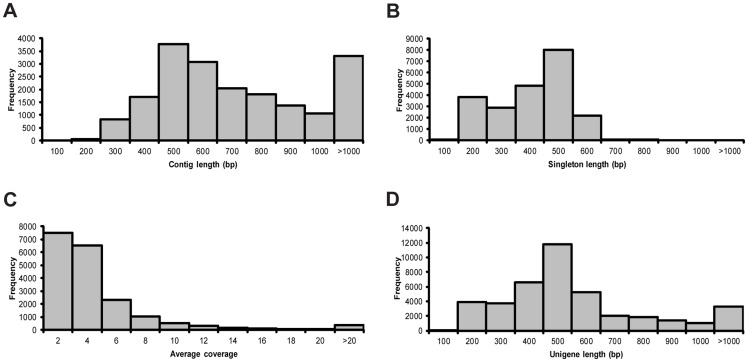
Assembly characteristics of *Cornus canadensis* transcriptome. (A) Length frequency distribution of assembled contigs. (B) Length frequency distribution of singletons. (C) Average coverage frequency distribution of assembled contigs. (D) Length frequency distribution of unigenes.

After the HQ reads from ccBud and ccLeaf were combined together for *de novo* assembly of transcriptome for *C. canadensis* (referred to as ccTranscriptome hereafter), a total of 19049 contigs (average coverage of five), 21835 singletons, and 40884 unigenes were obtained, with the average length of 714 bp, 358 bp and 524 bp, respectively (Table 2). The distributions of contig length, singleton length, average coverage of assembled contigs, and unigene length for ccTranscriptome are shown in Figure 3.

**Table 2:** Summary of assembly, BLAST and annotation for *Cornus canadensis* transcriptome.

	*Cornus canadensis*
Assembled contigs	19049
Mean of average coverage	5
Average length of contigs (bp)	714
Singletons	21835
Average length of singletons (bp)	358
Unigenes	40884
Average length of unigenes (bp)	524
Number of unigenes with BLAST matches; (% of total unigenes)	25891; 63.33%
Number of unigenes with GO annotation; (% of total unigenes)	19828; 48.50%

### Functional annotation

**Figure 4:**
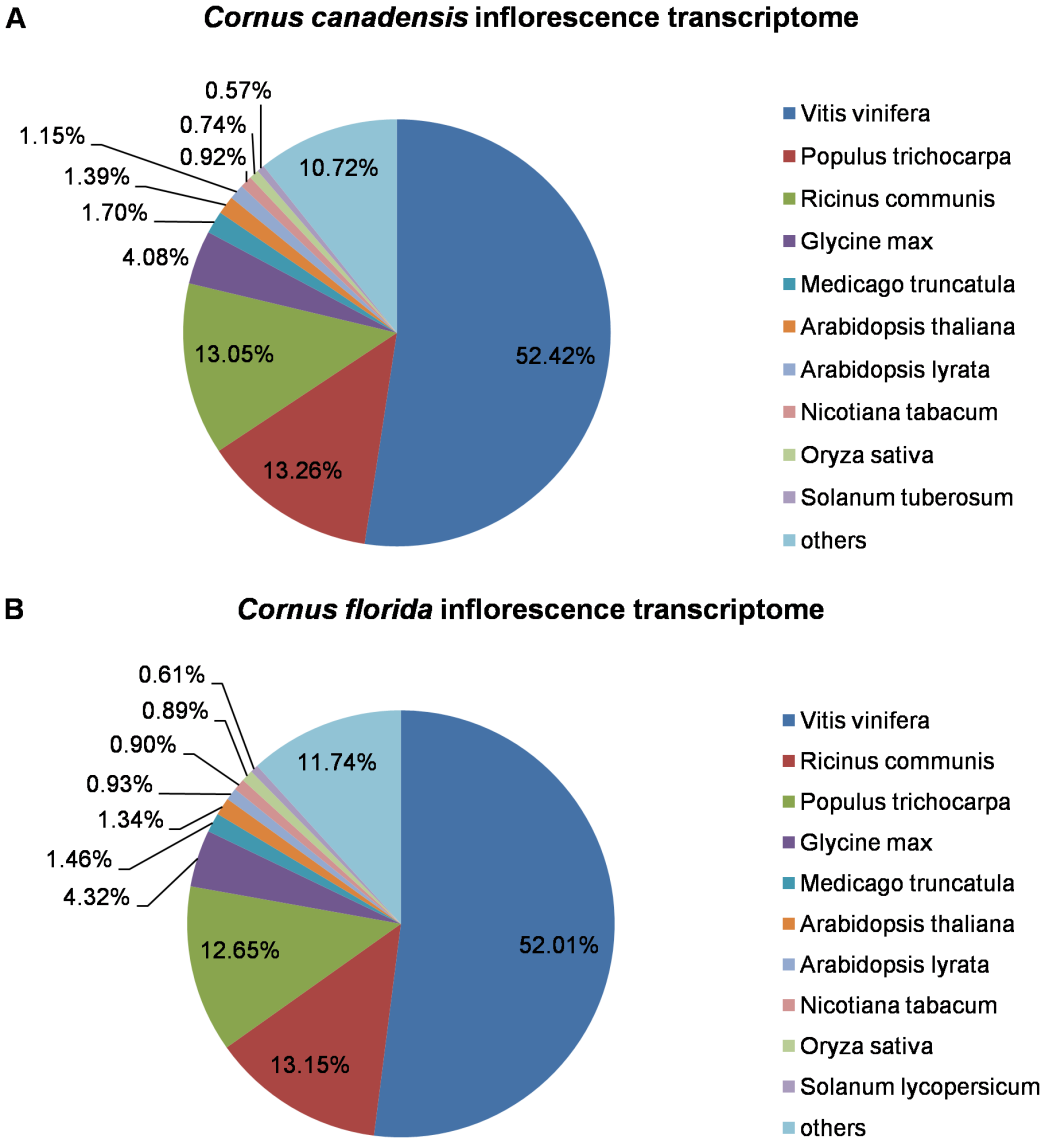
Top-hit species distribution of BLAST matches of two *Cornus* inflorescence transcriptome sequences. (A) *Cornus canadensis* and (B) *C. florida*.

**Figure 5:**
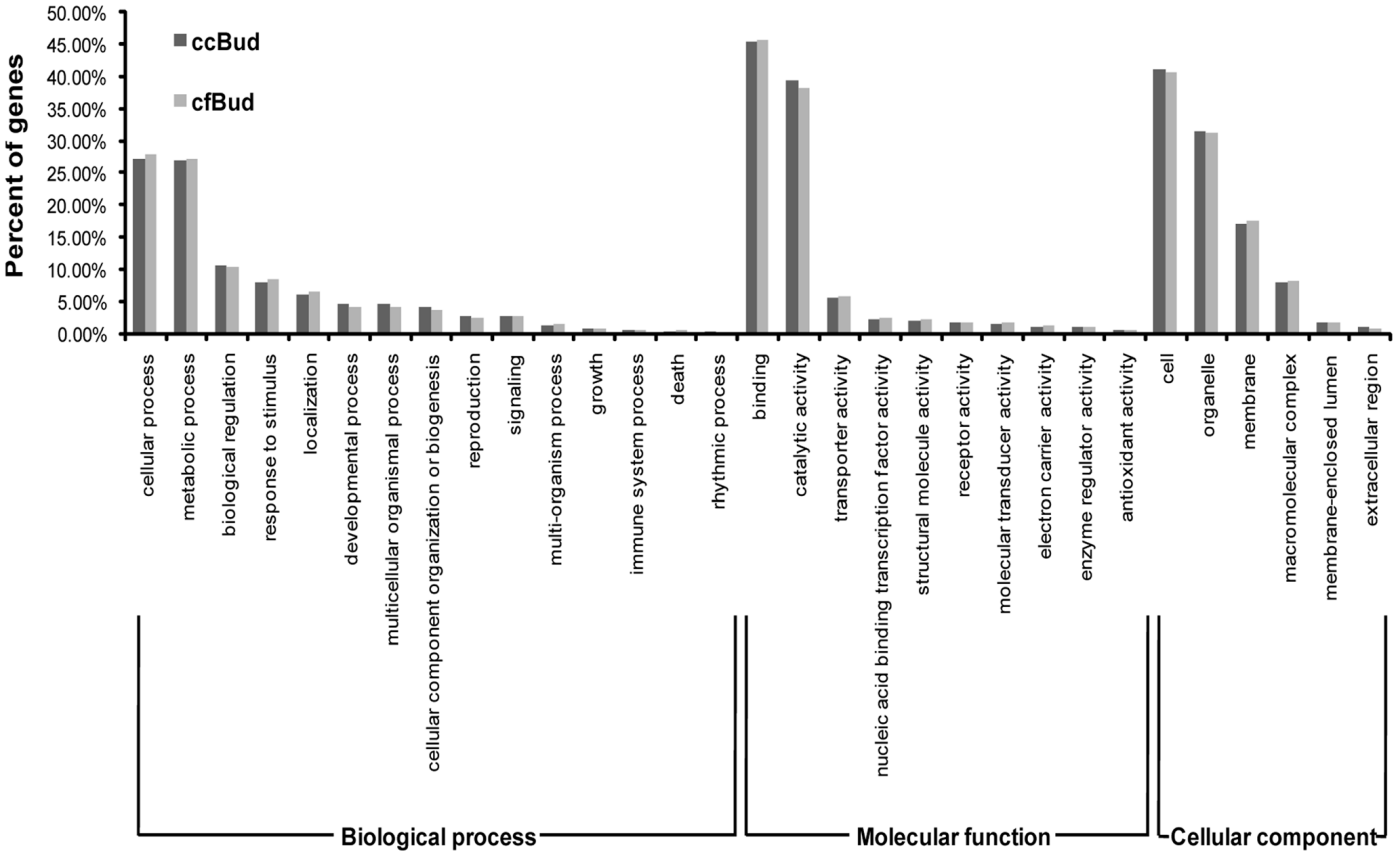
Gene ontology categories of unigenes in *Cornus canadensis* inflorescence (ccBud) and *C. florida* inflorescence (cfBud). The proportion of annotated unigenes from ccBud and cfBud classified into three gene ontology categories: biological process (32965 for ccBud vs 20163 for cfBud), molecular function (23111 for ccBud vs 14127 for cfBud) and cellular component (24486 for ccBud vs 15645 for cfBud).

After BLAST search, a total of 22231 (61.60%) unigenes from ccBud, 12434 (69.85%) from ccLeaf and 13723 (64.70%) from cfBud had significant BLAST matches (Table 1). For both *C. canadensis* and *C. florida*, the top three species of BLAST hit were *Vitis vinifera*, *Populus trichocarpa* and *Ricinus communis* (Figure 4). And 17229 (47.74%) unigenes from ccBud, 9728 (54.65%) from ccLeaf and 10694 (50.42%) from cfBud had at least one GO term assigned (some genes have more than one GO term) (Table 1). The distribution of GO terms was very similar between *C. canadensis* and *C. florida* inflorescence transcriptomes (ccBud and cfBud) (Figure 5). At the GO level 2, the annotation configuration found 32965 sequences assigned to biological process, 23111 to molecular function, and 24486 to cellular component. The corresponding numbers in cfBud were 20163, 14127 and 15645, respectively. In the biological process category, the most abundant sequences in both transcriptomes were classified to “cellular process” (GO: 0009987; 27.11% in ccBud and 27.79% in cfBud) and to “metabolic process” (GO: 0008152; 26.82% in ccBud and 27.19% in cfBud). In the molecular function category, sequences involved in “binding” (GO: 0005488; 45.49% in ccBud and 45.75% in cfBud) were highly represented, followed by sequences for “catalytic activity” (GO: 0003824; 39.41% in ccBud and 38.27% in cfBud). In the cellular component category, “cell” (GO: 0005623; 41.04% in ccBud and 40.59% in cfBud) and “organelle” (GO: 0043226; 31.50% in ccBud and 31.17% in cfBud) were the two most represented GO terms (Figure 5).

**Figure 6:**
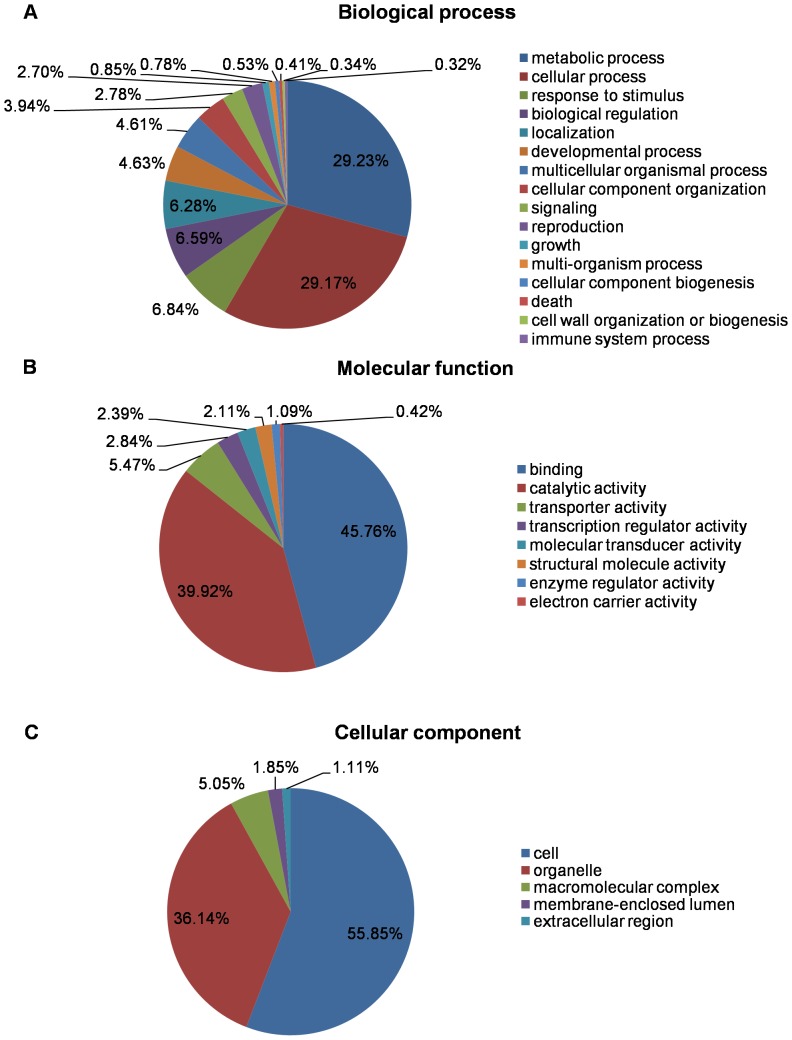
Gene ontology categories of unigenes in *Cornus canadensis* transcriptome. The proportion of annotated unigenes from ccTranscriptome classified into three gene ontology categories: (A) biological process, (B) molecular function and (C) cellular component.

Among the ccTranscriptome sequences, 25891 (63.33%) of them had significant BLAST matches, and 19828 (48.50%) were assigned to at least one GO term (also see Table 2). The distributions of most abundant GO terms for biological process (37752), molecular function (26128), and cellular component (22597) are presented in Figure 6. In the biological process category, the most abundant GO terms fall in the “metabolic process” (GO: 0008152) (29.23%) and “cellular process” (GO: 0009987) (29.17%), while the largest portion of sequences in the molecular function category was assigned to “binding” (GO: 0005488) (45.76%). In the cellular component category, the GO term “cell” (GO: 0005623) (55.85%) was highly represented (Figure 6).

### Comparative analysis

After reference mapping and BLAST search, there were 22057 consensus aligned sequences with BLAST hits for ccBud, 11206 for ccLeaf, and 8275 for cfBud. The *de novo* assembly of the unaligned HQ reads from cfBud yielded 14325 unigenes as the putative cfBud specific transcriptome (referred to as cfBud specific hereafter), with an average length of 408 bp (Table S1). The distributions of contig length, singleton length, average coverage of assembled contigs, and unigene length for cfBud specific transcriptome are shown in Figure S3. Among these unaligned unigenes of cfBud, 6979 (48.72%) sequences had BLAST matches (Table S1). Comparing all these results from BLAST search between ccBud and cfBud, we found that 7718 sequences with BLAST hits were shared by ccBud and cfBud, and 10926 only in ccBud and 6979 only in cfBud.

Furthermore, among these putative species-specific sequences, 7984 for ccBud and 5103 for cfBud could be assigned to GO terms. The annotation results showed that 180 ccBud-specific sequences were assigned to “flower development” (GO: 0009908) and 633 “response to stress” (GO: 0006950), while in cfBud-specific sequences, the numbers were 67 sequences related to “flower development” and 405 “response to stress”. Among these, the contigs with at least five mapped reads are shown in Table S2 for ccBud and Table S3 for cfBud. For example, the amino acid sequences of Ccanadensis_transcriptome_contig6848 (23 mapped reads and 215.16 RPKM value) and cfBud_transcriptome_contig966 (15 mapped reads and 464.94 RPKM value) in the “flower development” category were annotated as a member of basic helix-loop-helix (bHLH) DNA-binding superfamily proteins and an Ebs-bah-phd domain-containing protein, respectively (Table S2 and S3). The amino acid sequences of Ccanadensis_transcriptome_contig14719 (58 mapped reads and 465.54 RPKM value) and cfBud_transcriptome_contig2012 (77 mapped reads and 1705.30 RPKM value) in the “response to stress” category were annotated as a defensing protein and a major allergen, respectively (Table S2 and S3).

Among a total of 7718 sequences shared by ccBud and cfBud, putative DEGs between ccBud and cfBud were identified based on the RPKM values that are calculated from the read counts mapped onto the reference transcriptome (ccTranscriptome) and the corresponding unigene length. In these shared sequences, 6330 were annotated, including 943 with at least two-fold increase of RPKM value in ccBud than in cfBud, and 2513 with at least two-fold increase of RPKM value in cfBud than in ccBud. In “flower development” and “response to stress” categories, the putative DEGs with at least five mapped reads in ccBud or cfBud are listed in Table S4 and Table S5. To provide an example, in the “flower development” category, there were 66 ccBud reads mapped onto the Ccanadensis_transcriptome_contig3484 with 225.04 RPKM value while only two cfBud reads aligned onto this contig with 17.33 RPKM value (Table S4), which probably reflects a higher level of expression of the gene corresponding to contig3484 in ccBud. The putative protein of this contig was annotated to belong to the Enoyl-CoA hydratase/isomerase family (Table S4). In another example, in the “flower development” category, 13 cfBud reads were aligned onto the Ccanadensis_transcriptome_contig10032 with 261.48 RPKM value but only one ccBud read mapped onto the same contig with 7.92 RPKM value (Table S5). The putative protein of contig10032 was annotated to be a HUA enhancer 2 (Table S5).

Bi-directional BLAST searches between *Arabidopsis* and two dogwood transcriptomes showed that 87 (41.83% of 208) of *Arabidopsis* genes that regulate inflorescence architecture (27) and/or are expressed in inflorescence meristem (60) had putative orthologs in *C. canadensis* transcriptome and/or *C. florida* inflorescence specific transcriptome (Table S6). And some putative orthologs had greater RPKM values in ccBud than in cfBud (Table S6). Moreover, among the 87 genes with bi-directional best hits, 35 (40.23%) genes only had putative orthologs in *C. canadensis* inflorescence transcriptome, and 13 (14.94%) only had putative orthologs in *C. florida* inflorescence specific transcriptome (Table S6).

### SNP characterization

Mapping cfBud reads onto the ccTranscriptome identified 65931 high quality SNPs (excluding all gaps) from 2542 unigenes, including 2305 contigs and 237 singletons, with an average of 26 SNPs per unigene, between *C. canadensis* and *C. florida*. These predicted SNPs included 38385 transitions and 27546 transversions, at approximately a 1.4 : 1 ratio (Table 3). In addition, the frequencies between A/G and C/T transitions and frequencies among the four transversion types (A/T, G/T, C/G, A/C) were similar (Table 3). Among the unigenes containing SNPs, 2053 had annotation information, including 1983 contigs and 70 singletons. The information for the top 20 contigs containing relatively more SNPs is provided in Table S7. Taken the ccTranscriptome_contig4195 as an example, there was nearly equal read number from ccBud (33) and cfBud (34) mapped onto this contig, and 115 SNPs were found between the *C. canadensis* sequence and *C. florida* one, a proportion of 4.96% given the length of 2318 bp of the contig (Table S7). The annotation data showed that the putative protein of this contig was a homolog of a chloroplast heat shock protein (Table S7).

**Table 3:** Summary statistics of SNPs.

SNP type	Variations	Count number/Percentage
Transitions	A<->G	19041/28.9%
	C<->T	19344/29.3%
	Total	38385/58.2%
Transversions	A<->T	7697/11.7%
	G<->T	6840/10.4%
	C<->G	6130/9.3%
	A<->C	6879/10.4%
	Total	27546/41.8%

## Discussion

Our preliminary comparative sequencing of inflorescence transcriptomes in two *Cornus* species, *C. canadensis* and *C. florida*, using the Roche 454 GS-FLX method and *de novo* assembly generated abundant useful data. The sequencing produced HQ reads mostly 350–550 bp in length, and a great proportion of these (>85%) could be assembled into contigs that were longer than 500 bp (Figure S1 and Table 1). Although the average coverage of assembled contigs (mostly 5) was relatively low due to single sequencing run [24]–[26], the assembled ccTranscriptome of *C. canadensis* still provides the first reference transcriptome for *Cornus* species, offering a platform to perform comparative analysis to identify putative DEGs and interspecies SNPs for future evolutionary developmental biology and phylogenomic studies.

A large proportion of the unigenes had significant BLAST hits (61.60%, 69.85% and 64.70% of ccBud, ccLeaf and cfBud, respectively), and most of the best BLAST hits were plant proteins (Table 1 and Figure 4). However, a fraction of unigenes from our study had no significant matches to NCBI-NR database and TAIR database at the E-value threshold of 10^−6^. This phenomenon has also been reported in many other plants and the proportion of presumably unique sequences without BLAST hits were considered to be affected by species, sequencing depth, read length, and BLAST parameters [21], [27]–[29]. And “non-BLASTable” genes may include those rapidly evolving genes having homologs in other species but too divergent in *Cornus* to be recognized during BLAST. In addition, taxon-specific genes in the *Cornus* species that are missing from other databases may also contribute to the “non-BLASTable” category [21], [27]–[29].

### Identification of putative differentially expressed genes

Due to reasonably high cost of 454 sequencing, biological replicates were not included in the study, which prevented proper statistical testing on identification of DEGs. Nonetheless, annotated contigs abundant in one sample, rare or absent in the other still provide a valuable data source for selection of putative DEGs for analyses using qRT-PCR, *in situ* hybridization, and genetic transformation analyses (currently in progress in our lab) to evaluate their potential contributions to the inflorescence differences in *Cornus* (Table S2 and S3). For example, the putative ccBud specific contig, Ccanadensis_transcriptome_contig3886, is a homolog of *Arabidopsis ER* (*ERECTA*) gene (AT2G26330) (Table S2). A previous study in *Arabidopsis* suggested that loss of function in *ER* gene confers a corymb-like inflorescence due to a reduction in the length of stem internodes and pedicels [30]–[31]. Besides promoting inflorescence elongation, this gene was also found to regulate multiple developmental processes as well as environmental and biotic responses [32]–[33]. Therefore, we speculated that the absence of expression or the defect of function for these orthologous genes might contribute to the development of head-like and umbel-like compact inflorescence architecture in the big-bracted dogwoods (such as *C. florida*) and cornelian cherries (such as *C. officinalis*). Furthermore, among the 7718 unigenes shared by ccBud and cfBud, a number of these showed evident differences in RPKM values (Table S4 and S5). For instance, Ccanadensis_transcriptome_contig3484 had almost 13-fold increase of RPKM value in ccBud than in cfBud, and was annotated to be homologous to *AIM1* (*ABNORMAL INFLORESCENCE MERISTEM 1*) gene (AT4G29010) in *Arabidopsis* (Table S4). Mutation of *AIM1* gene was reported to affect inflorescence and floral development in *Arabidopsis* [34]. Another example is Ccanadensis_transcriptome_contig18209, which had nearly 19-fold increase of RPKM value in cfBud than in ccBud, and was identified to be a homolog of *ACL5* (*ACAULIS 5*) in *Arabidopsis* (Table S5). The *ACL5* gene is required for internodal elongation after flowering and its mutant exhibit a severe dwarf phenotype, with dramatically shortened inflorescence internodes and premature arrest of the inflorescence meristem [35]–[36], which is similar to the phenotype of *C. canadensis*. In addition, Ccanadensis_transcriptome_contig16216 was annotated to be a homolog of *WOX9* (*WUSCHEL-related homeobox 9*) with ten mapped-reads in ccBud and zero in cfBud (Table S2). In *Arabidopsis*, *WOX9* is required for meristem growth and maintenance [37]. In tomato, the homolog of *WOX9* gene (*COMPOUND INFLORESCENCE*, *S*) has been reported to determine inflorescence branching [38]. In the sympodial tomato, *s* mutant exhibited highly branched inflorescence [38], while in the monopodial *Arabidopsis*, *WOX9* RNAi constructs driven by a floral specific promoter also resulted in branching of floral meristems (Katie Liberatore, unpublished data). These data suggest a possible network of genes in regulating the development of inflorescences in *C. canadensis* and *C. florida*.

However, it must be noted that the total number of sequences identified to be specific to ccBud or cfBud via mapping onto ccTranscriptome reference might be overestimated in this study, due to the following reasons. First, the relatively low coverage might miss some low expressed genes in one of the two species, resulting in false identification of species-specific genes/transcripts. Second, in the cases where non-overlapping regions of the homologous gene sequences were recovered in cfBud and ccTranscriptomes, the alignment in reference mapping would have been failed, resulting in false identification of species-specific transcripts in cfBud. We investigated this by performing local BLAST search of randomly selected species-specific reads and found that some did match the same homologous gene sequences but aligned to the different regions of the genes. For example, the putative protein of cfBud_specific_singleton7973 that could not be aligned onto ccTranscriptome, matched the same protein sequence of *Arabidopsis* as that of ccTranscriptome_contig1425 did, indicating that the corresponding gene was not cfBud specifically expressed (Figure S4). This fact suggested that the non-overlapping regions of the homologous gene sequences recovered in ccTranscriptome and cfBud transcriptome can lead to the failure of the reference mapping for some cfBud sequences, therefore, resulting in false identification of some cfBud specifically expressed genes. Third, the potentially “high” divergence of homologous gene sequences between *C. canadensis* and *C. florida* due to phylogenetic divergence could also have led to false identification of cfBud specific transcripts. The two species have diverged >40 million years ago [12], [39]. However, if a sequence from cfBud of *C. florida* could be aligned to the homologous sequence from *Arabidopsis*, it would not fail to align to the homologous sequence from ccTranscriptome of *C. canadensis*, a congeneric sister lineage to *C. florida*, unless the sequences in each species are from the non-overlapping regions of the homologous gene sequences. Therefore, we rechecked the putative species-specific genes of interest, e.g., those listed in Table S2 and S3, to confirm that they were not falsely identified. The putative DEGs (including the species-specific ones and those shared by ccBud and cfBud, but with significantly different RPKM values in the two species) will serve as our best initial choices of candidate genes for analyses using qRT-PCR and *in situ* hybridization to characterize, in detail, their expression patterns in different dogwood species to evaluate their potential roles. Those displaying differences in expression pattern among species would then serve as the candidates for functional analyses through *in vivo* gene transformation using the systems established in *C. canadensis* [40], [41] and *Arabidopsis* [42].

### Orthologs of *Arabidopsis* inflorescence architecture related genes in dogwood inflorescence transcriptomes

We detected putative orthologs of most (27/41) of the reported inflorescence architecture related genes of *Arabidopsis* from the inflorescence transcriptomes of *C. canadensis* and *C. florida* (Table S6). These included some well-known regulators in flowering and inflorescence development, such as *SOC1*, *FUL*, *KNAT1*, *KNAT6*, *LFY*, etc (Table S6). This evidence suggests that the fundamental programs in flowering and inflorescence development may be conserved between *Arabidopsis* and dogwoods. In *Arabidopsis*, there were two major molecular programs controlling the inflorescence architecture. One regulates inflorescence internode elongation that includes the *ER*, *ACL5*, and *ACA10* genes among others. Orthologs of these genes regulating inflorescence internode elongation were also found in the inflorescence transcriptomes of both *C. canadensis* and *C. florida*, with some exhibiting differences in expression levels (Table S6). These genes might play a role in the divergence of inflorescence architecture between the dogwoods species, which can be tested by further investigation. The other program regulates inflorescence and floral meristem identity, which includes the well-known inflorescence architecture regulators, *LFY* (*LEAFY*), *TFL1* (*TERMINAL FLOWER 1*), and *AP1* (*APETALA1*). *LFY* and *AP1* were found to primarily promote floral meristem identity, while *TFL1* specify shoot identity and repress the floral meristem identity ([43] for a review). The *lfy* and *ap1* mutants caused delay of flowering and partial conversion of flowers into shoots or shoot-like structures, whereas *tfl1* mutant generated short inflorescences that terminate with a flower [44]–[51]. The orthologs of these genes and their inflorescence related functions have been reported in other plants, including tomato and petunia from the Asterids clade [38], [52]–[54]. We found *LFY* orthologous in our transcriptome data (Table S6), and *AP1* and *TFL1* orthologs in the inflorescence cDNAs of both species by gene cloning (unpublished data). These data similarly suggest that the key regulators on inflorescence and flower development of *Arabidopsis* have likely conserved their functions in *Cornus*. The failure in finding orthologs of *AP1* and *TFL1* in our transcriptome data was likely the result of low level expression or due to the limit of the sequencing depth. Our functional analysis of *CorTFL1* using *Agrobacterium*-mediated genetic transformation in *Arabidopsis* supported the function of *CorTFL1* from *C. canadensis* and *C. florida* in regulating flowering time and inflorescence development ([55]; unpublished data). However, whether these genes play a role in the divergence of inflorescence architecture among the dogwood lineages remain to be studied. Our recent investigation of *LFY* orthologs in *Cornus* (*CorLFY*) revealed no apparent difference in the expression pattern of *CorLFY* among different inflorescence types in both the early and late inflorescence developmental stages of multiple dogwood species [56]. It suggested that accumulation of transcripts of *CorLFY* might not contribute to the evolutionary changes of inflorescence architectures in the dogwood genus.

### Identification of SNPs for evolutionary study

We identified 65931 high quality SNPs between *C. canadensis* and *C. florida* sequences that were distributed among 2542 unigenes, with 26 SNPs per unigene. The level of sequence divergence between *C. canadensis* and *C. florida* is not particularly high, given that the two species have started to diverge in the Paleogene [4], [12]. Some examples of the SNP-containing genes found in this study are listed in Table S7. It is noteworthy that the SNP-containing genes identified in this study included some that have already been used as phylogenetic markers in other plant groups, such as the sucrose synthase gene (homologous to Ccanadensis_transcriptome_contig3878) which has been used in phylogenetic study of Leguminosae, and the eukaryotic translation elongation factor (homologous to Ccanadensis_transcriptome_contig3553) which has been used in phylogenetic study of eukaryotes [57]–[58]. The large number of SNP-containing genes identified in this study provides markers at the genomic scale to resolve phylogenetic relationships among dogwood species and their close relatives, e.g., for phylogenetic study of Cornaceae and Cornales, and also offers a source of candidate genes to perform ecological and evolutionary genetic study of adaptive traits in dogwood species, as done in other species [59]. Although the major clades within Cornales have been identified using conventional molecular markers (e.g., several chloroplast DNA genes and nuclear ribosomal genes) in previous studies, the relationships among the clades are still uncertain and/or controversial among different markers [1], [12], [39]. Therefore, it is expected that more nuclear gene data will be helpful to clarify the phylogenetic relationships among different clades. The SNP-containing genes permit designing primers for targeting amplification and sequencing of many different species simultaneously using the NGS platforms to generate markers for the analyses. However, it should be noted that the choice of a putative marker in this study requires orthologous comparison and variation assessment among species, which were not evaluated in our analyses.

## Conclusion

The data from comparative 454 sequencing of transcriptomes in *C. canadensis* and *C. florida* provide the first transcriptome information platform for functional genomics and evolutionary developmental biology studies in *Cornus*. The study identified meaningful candidate genes for future analyses to understand the genetic mechanisms underlying dogwood inflorescence evolution. Furthermore, the study generated a wealth of potential genetic markers useful for genetic mapping, phylogenetic and population genetic studies.

## Materials and Methods

### RNA extraction and cDNA synthesis

The inflorescence buds of *C. canadensis* (ccBud) and *C. florida* (cfBud), as well as young and mature leaves of *C. canadensis* (ccLeaf), were collected from living plants and stored in RNAlater (Ambion of Applied Biosystems, Foster City, CA, USA) immediately after removing from plants. Materials were stored at −20°C until total RNA extraction. Total RNAs were extracted from tissues pooled from two or more plants of the same species using the modified CTAB RNA isolation method [60]. The unnormalized cDNAs of ccBud and cfBud were synthesized and processed into sequencing libraries according to Roche's cDNA Rapid Library Preparation Method and standard Rapid Library kit (cat# 05 608 228 001). The cDNAs of ccLeaf were first synthesized using Evrogen's (Moscow, Russia) MINT-Universal cDNA synthesis kit (cat# SK005), normalized using the Evrogen Trimmer kit (cat# NK003), and then processed into a sequencing library using Roche's standard Rapid Library kit (all procedures following manufacturer's recommendations). Concentration and quality of the libraries were assayed using Agilent's (Santa Clara, CA, USA) Bioanalyzer.

### 454 sequencing and assembly

The rapid library adapters A and B were ligated on the cDNA samples, and then the three cDNA libraries (ccBud, ccLeaf and cfBud) were subjected to clonal amplification by emulsion PCR. The clonally amplified beads of the three samples were enriched and sequenced by a half-plate run on the 454 GS-FLX Titanium platform following manufacturer's protocol (Roche Diagnostics, USA). Roche's onboard base caller was used to generate files that contain base and quality information for raw reads. The raw reads produced in this study have been deposited in the DNA Data Bank of Japan (DDBJ) Sequence Read Archive (DRA) (accession number: DRA001182). Before assembly, the low quality sequences, ambiguous nucleotides, adapter sequences, short sequences (<50 bp) and 454 sequence primers were removed from the raw reads through data trimming using CLC Genomics Workbench 4.6.1 (CLC Bio, Aarhus, Denmark). The high-quality (HQ) reads from ccBud, ccLeaf and cfBud were assembled *de novo* individually. Furthermore, the HQ reads from ccBud and ccLeaf were pooled together for *de novo* assembly of *C. canadensis* transcriptome (ccTranscriptome), which was used as a reference transcriptome for following analyses. The *de novo* assembly was generated using CLC Genomics Workbench 4.6.1 with default parameters, e.g., a minimum length fraction of 0.9, minimum similarity fraction of 0.8, maximum number of two mismatches, and minimum contig length of 100. The HQ reads with ≥100 bp length that could not be assembled into contigs were remained as singletons. The contigs and singletons were defined as unique sequences (also referred to as unigenes). In the following comparative analyses, the HQ reads from ccBud, ccLeaf and cfBud were used to map onto the reference sequences, ccTranscriptome in CLC Genomics Workbench 4.6.1 with the long reads mapping parameters, mismatch cost of 2, insertion cost of 3, deletion cost of 3, length fraction of 0.5, and similarity of 0.8 [61]–[62]. The portion of HQ reads from cfBud that could not be mapped onto the reference transcriptome (ccTranscriptome) (referred to as “cfBud specific”) were assembled *de novo* using CLC Genomics Workbench 4.6.1 with default parameters described above.

### BLAST and annotation

The contigs and singletons from all *de novo* assembled transcriptomes were compared to NCBI non-redundant (nr) protein database (NCBI-NR database) and Arabidopsis Information Resource (TAIR) database (TAIR10) using BLASTx algorithm with the E-value threshold of 10^−6^, in order to find the corresponding homologous genes from other species [63]. The Gene Ontology (GO) annotation was performed using Blast2GO program with the annotation cut-off of 10^−6^ [64]–[65]. The best five protein hits for each query were parsed out to create annotated tables, containing available information such as taxonomy, protein function, accession number, etc. And the best blast hits were used to retrieve associated GO terms describing biological process, molecular function, and cellular component [64]. In order to get the information on general functional categories, the final GO graph was generated, which summarized the distribution of the GO level 2 terms for three main categories (biological process, molecular function and cellular component) [26].

### Comparative analysis of transcriptomes

The HQ reads from ccBud, cfBud, and ccLeaf transcriptomes were mapped onto the reference transcriptome (ccTranscriptome). After the reference mapping, the alignment was visualized by Integrative Genomics Viewer (IGV) [66]. The number of reads mapped onto each unigene and the length of each unigene were exported into excel table for expression comparison. The relative expression levels for unigenes were assessed by Reads per Kilobase per Million mapped Reads (RPKM), using the formula, R(G) = (10^9^*C)/NL, C = number of reads mapped to unigene G, N = number of reads mapped in each library, L = length of unigene G [67]. A RPKM value with at least two-fold difference between the two samples was used as criteria to determine putative differentially expressed genes (DEGs) [68]–[69]. The contigs and singletons of ccTranscriptome that only had mapped reads from ccBud were treated as putative specific expressed genes of ccBud. The *de novo* assembled contigs and singletons from unmapped reads of cfBud (cfBud specific) were treated as putative specific expressed genes for cfBud. Furthermore, to search whether inflorescence regulators found in model plants are present in our transcriptome data, we selected 41 inflorescence architecture related genes reported in *Arabidopsis* and 167 inflorescence meristem expressed genes in *Arabidopsis* for local tBLASTn (blast-2.2.28+ (ftp://ftp.ncbi.nlm.nih.gov/blast/executables/blast/LATEST/)) search against the assembly data from ccTranscriptome and cfBud specific (as local BLAST databases) with the E-value threshold of 10^−6^, and then the best BLAST hits from our local databases were used as queries to do BLASTx against TAIR10 with the E-value threshold of 10^−6^. Through this bi-directional BLAST [70]–[74], the evolutionary orthologs between *Arabidopsis* and two dogwood species were found (Table S6),

### SNP discovery

In order to detect interspecies single nucleotide polymorphisms (SNPs) between *C. canadensis* and *C. florida*, we used ccTranscriptome assembled from ccBud and ccLeaf HQ reads as a reference, and mapped the HQ reads from cfBud onto the reference transcriptome through CLC Genomics Workbench 4.6.1. A SNP was called when all *C. florida* reads produced a consensus base that was different from that in the *C. canadensis* reference. Following recent publications [75]–[76], we called a SNP when it was supported by at least three reads and all the reads agreed on the same consensus base calls. The SNP sites between ccBud and cfBud could be visualized by IGV.

## Supporting Information

Figure S1 **Length distribution of raw reads and high quality (HQ) reads.** (A) ccBud, (B) cfBud and (C) ccLeaf.
(TIF)

Figure S2 **Assembly characteristics of** ***Cornus canadensis*** **leaf transcriptome (ccLeaf).** (A) Length frequency distribution of assembled contigs. (B) Length frequency distribution of singletons. (C) Average coverage frequency distribution of assembled contigs. (D) Length frequency distribution of unigenes.
(TIF)

Figure S3 **Assembly characteristics of** ***Cornus florida*** **inflorescence specific transcriptome (cfBud specific).** (A) Length frequency distribution of assembled contigs. (B) Length frequency distribution of singletons. (C) Average coverage frequency distribution of assembled contigs. (D) Length frequency distribution of unigenes.
(TIF)

Figure S4 **An example of protein alignment for local BLAST results.** The protein sequences in the alignment were translated from Ccanadensis_transcriptome_contig1425, cfBud_transcriptome_singleton7973 and *LFY* of *Arabidopsis*, a query gene.
(TIF)

Table S1 **Summary of assembly, BLAST and annotation for** ***Cornus florida*** **specific sequences.**
(DOC)

Table S2 **Examples of sequences specific to** ***Cornus canadensis*** **transcriptome.**
(XLS)

Table S3 **Examples of sequences specific to** ***Cornus florida*** **transcriptome.**
(XLS)

Table S4 **Examples of sequences with higher RPKM value in** ***Cornus canadensis*** **than in** ***C. florida*** **.**
(XLS)

Table S5 **Examples of sequences with higher RPKM value in** ***Cornus florida*** **than in** ***C. canadensis*** **.**
(XLS)

Table S6 **Putative dogwood orthologs of** ***Arabidopsis*** **genes involved in inflorescence architecture regulation identified by bi-directional BLAST.**
(XLS)

Table S7 **Top 20 contigs with most SNPs between** ***Cornus canadensis*** **and** ***C. florida*** **inflorescence transcriptomes.**
(XLS)
