# Supplementary figures and images for: De novo Sequencing, Characterization, and Comparison of Inflorescence Transcriptomes of Cornus canadensis and C. florida (Cornaceae)
Source: PLoS One. 2013 Dec 27;8(12):e82674. doi: 10.1371/journal.pone.0082674 (PMC3873919; doi:10.1371/journal.pone.0082674)

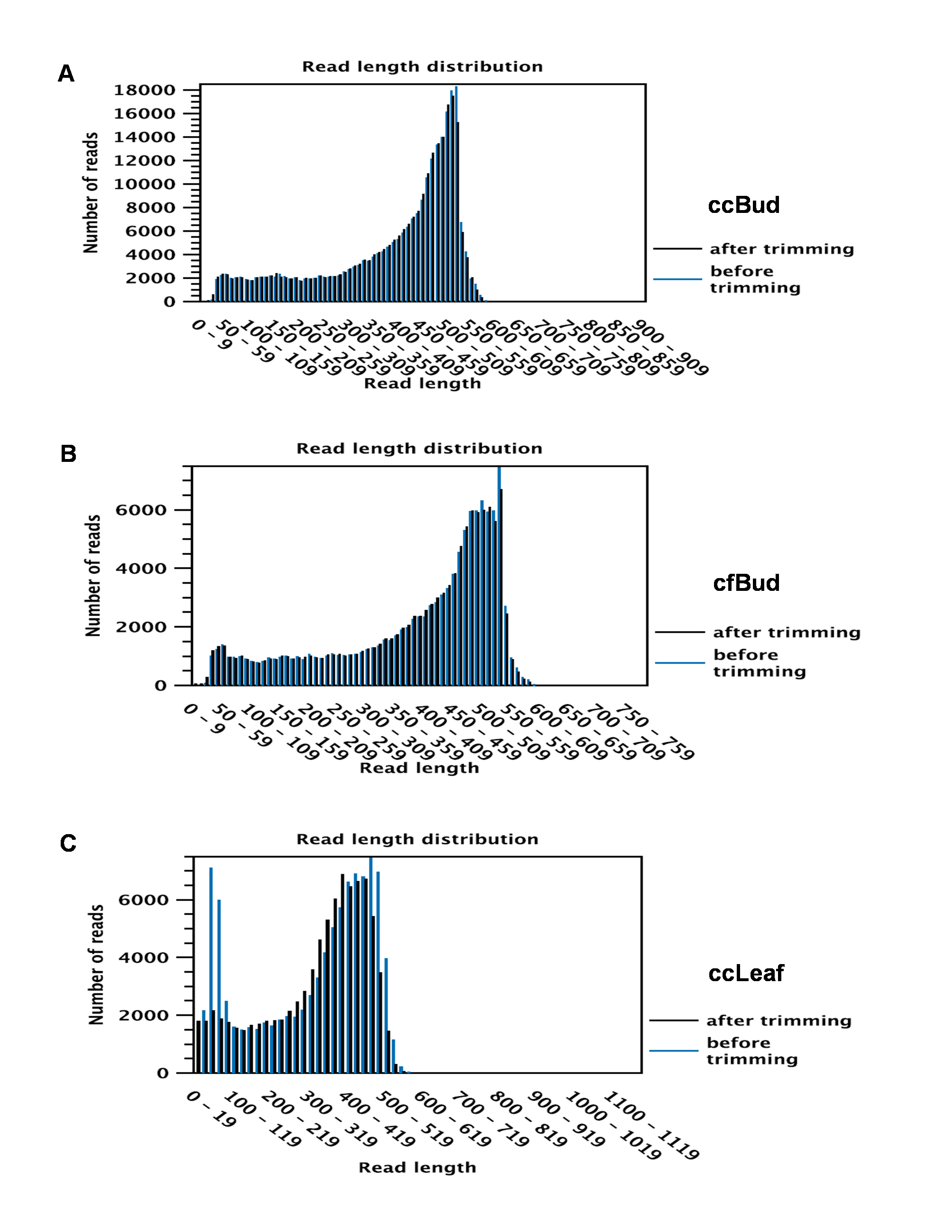

Supplement: Figure S1 — Length distribution of raw reads and high quality (HQ) reads. (A) ccBud, (B) cfBud and (C) ccLeaf. (TIF) [file pone.0082674.s001.tif]

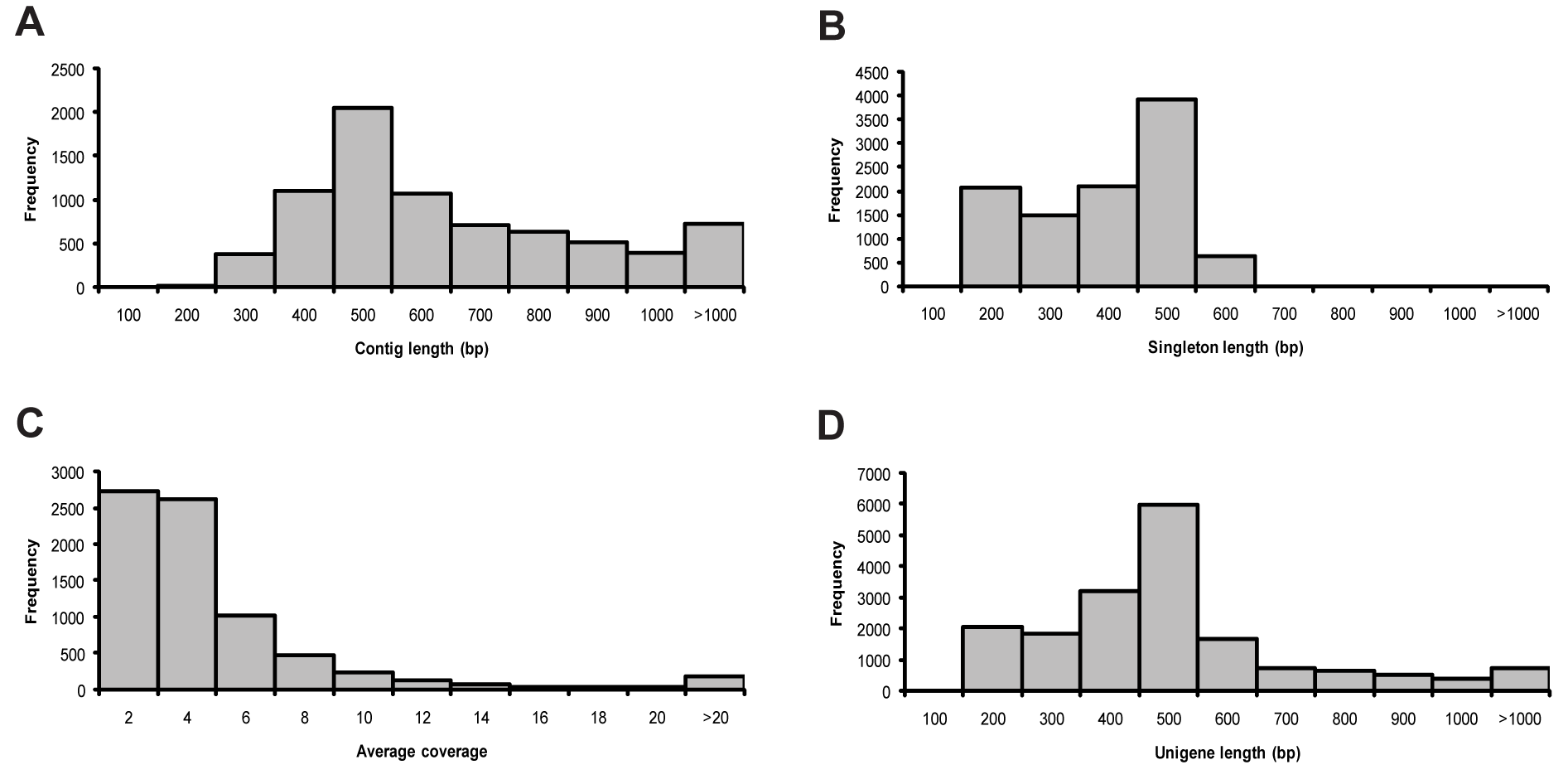

Supplement: Figure S2 — Assembly characteristics of Cornus canadensis leaf transcriptome (ccLeaf). (A) Length frequency distribution of assembled contigs. (B) Length frequency distribution of singletons. (C) Average coverage frequency distribution of assembled contigs. (D) Length frequency distribution of unigenes. (TIF) [file pone.0082674.s002.tif]

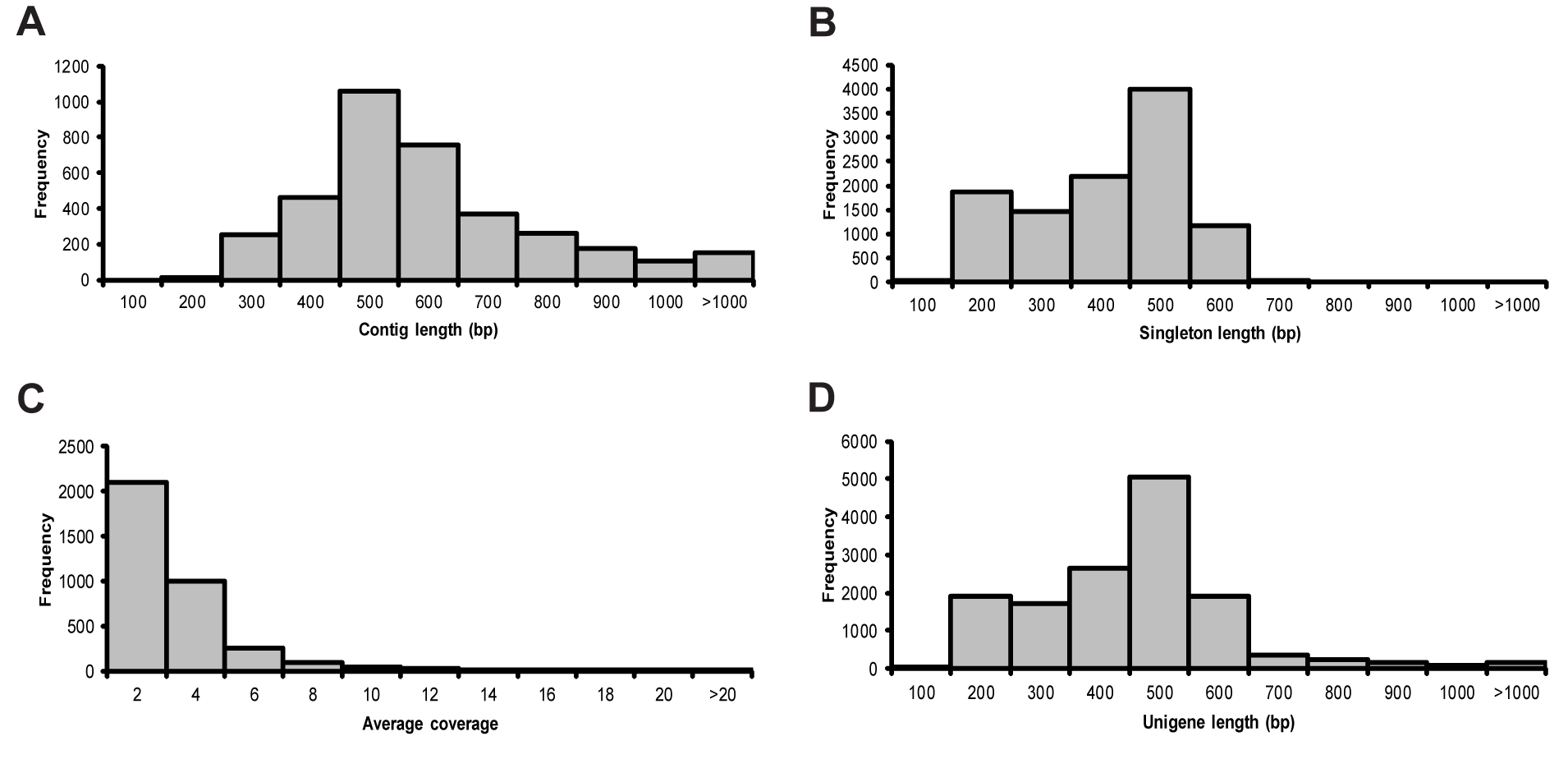

Supplement: Figure S3 — Assembly characteristics of Cornus florida inflorescence specific transcriptome (cfBud specific). (A) Length frequency distribution of assembled contigs. (B) Length frequency distribution of singletons. (C) Average coverage frequency distribution of assembled contigs. (D) Length frequency distribution of unigenes. (TIF) [file pone.0082674.s003.tif]

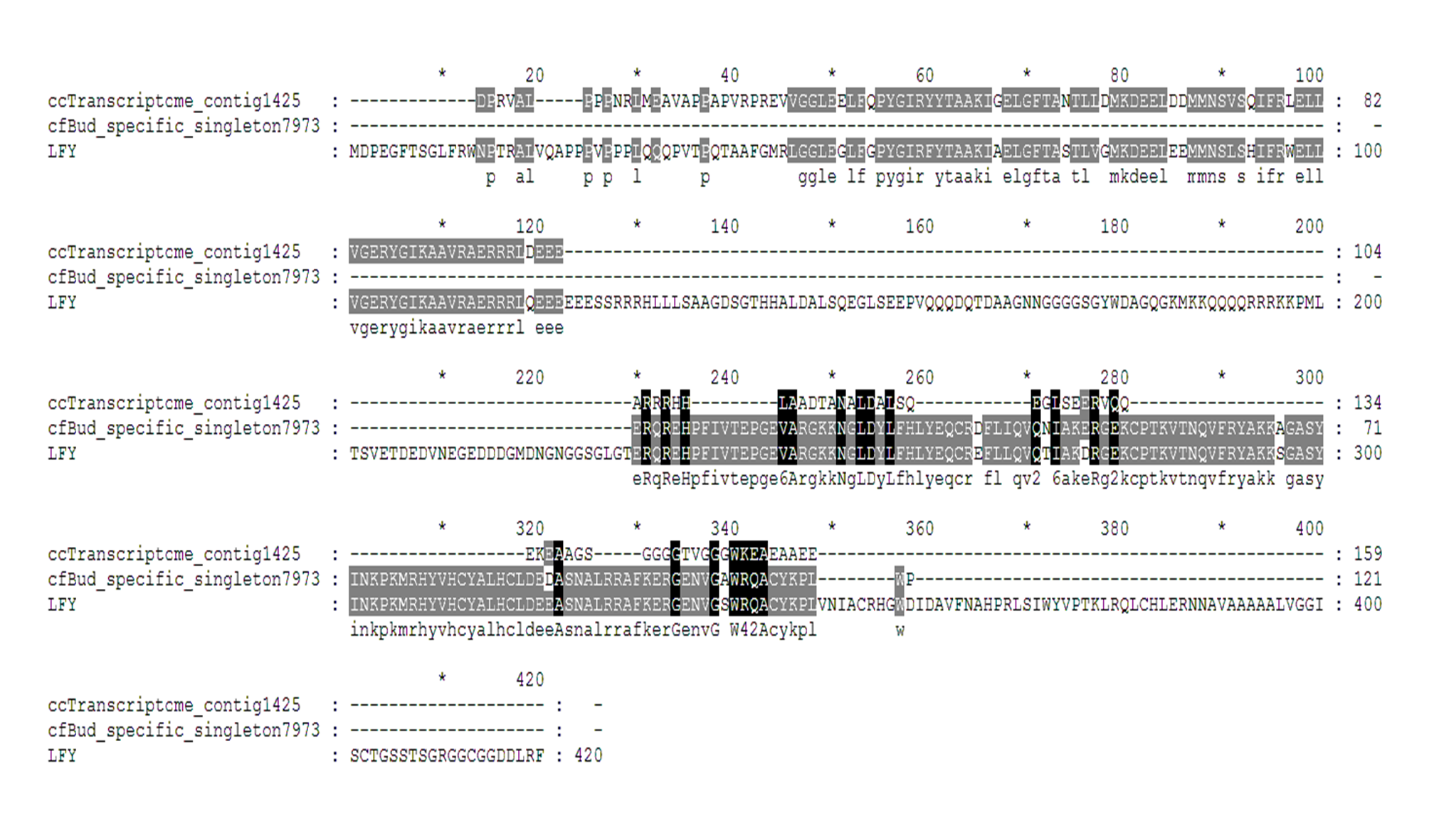

Supplement: Figure S4 — An example of protein alignment for local BLAST results. The protein sequences in the alignment were translated from Ccanadensis_transcriptome_contig1425, cfBud_transcriptome_singleton7973 and LFY of Arabidopsis, a query gene. (TIF) [file pone.0082674.s004.tif]
