# Supplementary material for: De novo Sequencing, Characterization, and Comparison of Inflorescence Transcriptomes of Cornus canadensis and C. florida (Cornaceae)
Source: PLoS One. 2013 Dec 27;8(12):e82674. doi: 10.1371/journal.pone.0082674 (PMC3873919; doi:10.1371/journal.pone.0082674)
Supplement: Table S1 — Summary of assembly, BLAST and annotation for Cornus florida specific sequences. (DOC) [file pone.0082674.s005.doc]

**Table S1. Summary of assembly, BLAST and annotation for *Cornus florida* specific sequences.**

|  | ***Cornus florida*** |
| --- | --- |
| Assembled contigs | 3593 |
| Mean of average coverage | 3 |
| Average length of contigs (bp) | 553 |
| Singletons | 10732 |
| Average length of singletons (bp) | 360 |
| Unigenes | 14325 |
| Average length of unigenes (bp) | 408 |
| Number of unigenes with BLAST matches  (% of total unigenes) | 6979  48.72% |
| Number of unigenes with GO annotation  (% of total unigenes) | 5103  35.62% |
